# Wired Emotions: Ethical Issues of Affective Brain–Computer Interfaces

**DOI:** 10.1007/s11948-019-00087-2

**Published:** 2019-03-13

**Authors:** Steffen Steinert, Orsolya Friedrich

**Affiliations:** 1 Department of Values, Technology and Innovation, Faculty of Technology, Policy and Management, Delft University of Technology, Delft, The Netherlands; 2 Institute of Ethics, History and Theory of Medicine, Ludwig-Maximilians-Universität München, Lessingstr. 2, 80336 Munich, Germany

**Keywords:** Affective brain–computer interface, Emotion, Brain–computer interface, Affective states

## Abstract

Ethical issues concerning brain–computer interfaces (BCIs) have already received a considerable amount of attention. However, one particular form of BCI has not received the attention that it deserves: Affective BCIs that allow for the detection and stimulation of affective states. This paper brings the ethical issues of affective BCIs in sharper focus. The paper briefly reviews recent applications of affective BCIs and considers ethical issues that arise from these applications. Ethical issues that affective BCIs share with other neurotechnologies are presented and ethical concerns that are specific to affective BCIs are identified and discussed.

## Introduction

Research on brain–computer interfaces (BCIs) is flourishing and is attracting more and more attention and investment. For example, in 2016 the well-known entrepreneur Elon Musk co-founded the neurotechnology company Neuralink that aims to create BCIs, and Facebook has a secret hardware project that works on BCIs (Marsh 2018). Brain–computer interfaces have a wide range of application by enabling disembodied agency, that is acting without moving the body (Steinert et al. 2018). Affective BCI is a technology that is able to detect, influence and stimulate affective states. Whereas brain–computer interfaces in general have already received a fair amount of ethical and theoretical treatment, the sub-field of *affective* brain–computer interfaces has not yet received the ethical scrutiny that it deserves. This paper seeks to close this gap.

A few clarifying remarks: Affective states are experiential phenomena like emotions and moods. Emotions are intentional mental states because they involve a relation between the person and something else (i.e., the object of the emotion). For example, one is angry with someone or afraid of something. Further, emotions involve evaluations of something, emotions are usually accompanied by bodily feelings, and emotions are motivational. In contrast, moods are usually long-term, not intentional and more diffuse.

Affective states are important because they are closely linked to values and emotions, particularly, play a crucial role in moral judgment (Roeser and Todd 2014). Further, emotions play a central role in human life, as they are important in interpersonal relationships, contribute to group formation and play a role in decision making and reasoning. Because affective states are one of the essential ways in which humans engage with the world it is critical to accompany the development of affective BCIs with ethical reflection as early as possible.

### Affective BCIs: Recent Trends and Applications

What are affective BCIs and what are they used for? Affective BCIs work like other brain–computer interfaces in that they read out neural signals that are then used to perform a certain task (Mühl et al. 2014). An affective BCI is a system that uses neurophysiological signals to extract features that are related to affective states (e.g., emotions and moods). Brain signals can be measured invasively or non-invasively. Invasively means that electrodes are inserted into the body. One example of an invasive method is electrocorticography (ECoG) where electrodes are placed on the surface of the brain in order to measure the activity of the cerebral cortex. In contrast, non-invasive neurotechnology measures the brain activity from outside the head. For instance, electroencephalography (EEG) uses electrodes that are placed on the surface of the skull. Another non-invasive method to monitor brain activity is functional near-infrared spectroscopy (fNIRS) where near-infrared light is used to pick up on changes in the brain’s blood oxygen level that are linked to brain activity. The output signals can be used as feedback to the user or as input for computer systems, or both. Accordingly, the detection of affective states via affective BCI can be used to modify human–computer interaction. Affective BCIs may be located within the field of affective computing that, among other things, seeks to utilize information about affective states to enhance the interaction with computers (Picard 2000). Of course, affective BCIs are not the only way to detect affective states. It is also possible to utilize physiological (e.g., blood pressure) or behavioral (e.g., facial recognition) signals instead of neurophysiological signals, or even combine different modalities to enhance classification accuracy (Chanel et al. 2009).

It is worth pointing out here that research on affective BCI is an emerging field and current affective BCI technology cannot smoothly be applied to most real-world contexts yet. While mapping and detecting emotions via EEG is difficult, a lot of advances in the area of classifying discrete emotions (e.g., fear, surprise, disgust) have recently been made (Bono et al. 2016; Lee and Hsieh 2014). These advances have prompted some authors (e.g., Lin et al. 2015, 319) to express confidence that affective BCI systems for everyday use are feasible in the near future. So, while some of the applications considered in the paper are to a certain extent speculative, they nevertheless give us a glimpse of what will (sooner or later) be possible.

In recent years, there have been some major advances in the technological ability to recognize affective states. For example, Wu et al. (2017) report a novel method involving EEG that recognizes negative and positive emotional states with high accuracy. The authors propose that their method could be used in wearable EEG systems that monitor emotions on an everyday basis. The accurate detection of emotions could be utilized in other areas as well. For instance, Ali et al. (2016) suggest that their EEG-based approach to emotion detection can be helpful in the context of healthcare, e.g. in ambient assisted living facilities.

Besides detecting affective states, it is also possible to use affective brain–computer interfaces to stimulate and influence the affective states of people. Daly et al. (2016) developed an affective BCI system that can detect the current affective state and modulate it by playing emotionally evocative music, thereby moving people from one affective state to another. For example, participants could be moved from a neutral state to feeling happy or from an excited state to a calm state. Other researchers also used music combined with affective BCI systems to influence the affective state of the subjects (Ehrlich et al. 2017).

When there is a continuous interaction between brain–computer interface systems and brain activity this is called a closed-loop system. Another area where affective brain–computer interfaces have been said to be helpful is in the deep brain stimulation of the limbic circuit of people with emotional disorders. For example, a closed-loop system comprised of an emotion decoder and a stimulation device could serve as an ‘emotional prosthesis’ (Widge et al. 2014). Such an emotional prosthesis could be used to ameliorate the painful memories of traumatic events.

Affective BCIs can also facilitate emotion expression. In particular, patients with severe motor impairments, like amyotrophic lateral sclerosis (ALS), which is a group of neuronal diseases that mainly causes the degeneration of neurons that control voluntary muscle movements, find it hard to express their emotions (Kashihara 2014). Affective BCIs can give patients the opportunity to express their emotions, thereby increasing their quality of life (Nijboer et al. 2009).

Affective BCI technology need not be limited to therapeutic applications, the medical context and scientific research. Andujar et al. (2015) hypothesize that an affective BCI could also be helpful in non-face-to-face communication by displaying the emotional status of the communication partner. Further, a wearable device (e.g., bracelets or rings) could inform the wearers, and others, that they are in a particular affective state (Hao et al. 2014). Thereby, an affective BCI may help one to express affective states in a non-conventional way. Similarly, a way to broadcast people’s affective states via affective BCI are so-called artistic BCIs, in which the affective state of the user is influenced (e.g., by sound or image) and then represented “[…] visually or through a type of audio where the corresponding user and others are able to perceive visually or audibly how the user is feeling.” (Andujar et al. 2015, 62).

Affective BCIs could also be used in the entertainment sector. For example, Brouwer et al. (2015) present an affective BCI system that picks up the affective states of the users while they are reading a novel. Based on the changing affective states during reading, the system provides a particular version of the section of the novel. Further, levels of frustration or joy could be used to adapt a computer application to the affective state of the user. Based on research on the classification of sadness and happiness using EEG (Pan et al. 2016) and research on the neurophysiological underpinnings of frustration (Myrden and Chau 2017; Reuderink et al. 2013), one can easily envision a computer application that adapts to these affective states of the user. A potential field for such adaptive computer applications is computer games, where information about the affective state of the user could be used to change how the game is presented or how the game unfolds in order to match or influence the affective state of the player (Andujar et al. 2015). This means that the game will be more individualized to fit the respective player. Everybody would be playing a different game.

Some consumer products that utilize affective states are already on the market. For instance, *Mico*, developed by the Japanese company Neurowear, is a headphone that selects music based on the mood of the wearer. Further, *Neurocam*, by the same company, is a wearable camera that detects the emotions of the user and automatically takes a snapshot in moments where the user is emotionally engaged (Neurowear 2018).^1^ A domain where affective BCIs have already been applied is in the music industry. For instance, an affective BCI has been successfully used to measure the affective states of the listeners, also of the performer during a live performance and to make the system adapt to each respective affective states (Eaton et al. 2015), thus harmonizing the affects. Furthermore, detecting the listener’s affective state may enable individualized pieces of music, as the system can adapt to the affective state in real-time. Other possible applications for affective BCIs regarding music are described by Andujar et al. (2015).

### Affective BCIs and Ethical Issues

The studies referenced above provide ample indication that highly sophisticated forms of detecting affective states are feasible. As emotions play a vital part in people’s lives and are a crucial aspect of what it means to be human, the ethical implications of these developments should be reflected. Of course, not all of the ethical issues that arise in connection with affective BCIs are completely new. There are some ethical issues, like harm-benefit evaluations and how to deal with the collection of sensitive data, that affective BCIs share with similar neurotechnologies, particularly other types of BCIs. These ethical issues will be briefly addressed in this section and the main focus of the remainder of the paper is on the unique ethical challenges that are raised by affective BCIs. These challenges have to do with the capabilities of affective BCIs to monitor, influence and directly stimulate the affective states of people. The table below encapsulates the ethical issues that affective BCIs have in common with other forms of BCI and the ethical challenges specific to affective BCIs (Table 1).

**Table 1:** Ethical issues of affective BCIs

Common ethical issues of affective and other BCIs	Specific ethical issues related to affective BCIs		
	Monitoring of affective states	Influencing affective states	Directly stimulating affective states
Risk to the body (e.g., infections, damage to tissue) Evaluation benefit—harm Data security and privacy Potentially false expectations Informed consent Problems of shared control, criminal guilt and liability Impact on self, agency, identity and personhood (e.g. through self-quantification) Biases embedded in the device	Self-tracking of emotions could infringe on autonomy and authenticity Fostering of emotion-stereotypes Alienation from one’s own emotions Social pressure to self-regulate or enhance control over emotions	Manipulation of affective processes and thereby of intentions, decisions, actions Novel threats to mental integrity and cognitive liberty New ways of nudging/emotional influence by companies or government Issues with living in an automatically emotion-adjusted environment Outsourcing of emotion regulation Responsibility for emotions Questions of what it means to be human	In closed-loop systems: issues of emotional self-regulation and responsibility Non-authentic emotions Undermining sense of self, agency and self-determination Potential self-estrangement when emotions conflict with judgment Problems in assessing the origin of an emotion Psychological distress as a harm Changes in autobiographic memory, sense of self, identity Issues of personhood Responsibility ascription (e.g., manipulation of emotions in military context)

Affective BCIs share certain ethically relevant issues, like risks to the body, data protection and informed consent, with other neurotechnologies. Affective BCIs can take an invasive form, where the technology is embedded in the brain. Here there is the risk of infection or brain tissue injuries. Because the avoidance of harm is a basic value in medical ethics, the well-being of the patient, the benefits of the procedure and the potential harm of the intervention need to be balanced carefully. So, similar to other invasive neurotechnologies, the ethical evaluation of benefit and harm is crucial when it comes to the use of invasive BCIs (Glannon 2014, 2016), and invasive affective BCIs are no exception here.

When affective BCIs are deployed in a medical or research context, two issues that need to be addressed are the management of expectation and informed consent (Klein 2016; McCullagh et al. 2014; Vlek et al. 2012). A person’s self-determination is an important ethical value and a person needs to understand the potential risks of every medical intervention before consenting to the procedure. Understanding the (long-term) consequences of detecting, influencing and stimulating affective states via affective BCIs can be difficult and therefore, the process of informed consent requires particular attention.

All BCI systems collect sensitive data, which is why the issues of data security, privacy and neuro-hacking need to be addressed (Attiah and Farah 2014; Ienca and Haselager 2016; Jebari 2013; Klein 2016; O’Brolchain and Gordijn 2014). These issues also need to be tackled when it comes to affective BCI because affective BCIs collect data about affective states, which is a very sensitive topic for most people. Data about affective states belong to an individual’s personal data and therefore need to be protected from any undue treatment by other parties. Given that affective BCI systems will also include elements that are not fully under the control of the user, there are some well-known concerns, like shared control and criminal guilt, that have already been addressed concerning other BCI applications (Grübler 2011; Lucivero and Tamburrini 2008; O’Brolchain and Gordijn 2014; Tamburrini 2009; Weinberger and Greenbaum 2016). Recently, researchers have called for a veto control for semi-autonomous BCI systems (Clausen et al. 2017). This type of veto control also seems to be something that is worth thinking about regarding affective BCI systems. At the very least, users of affective BCIs should be enabled to understand what the system does and why, and what kind of data are collected and processed.

#### Affective BCIs and Monitoring of Affective States

In addition to the ethical concerns shared with other neurotechnologies, there are several ethical challenges that are unique to affective BCIs by virtue of their potential to monitor, influence and stimulate affective states. Some of these ethical issues, for example, autonomy (Friedrich et al. 2018), have already been addressed in the literature on other BCIs. Nevertheless, these ethical issues are important for affective BCIs as well and will be briefly addressed where necessary.

There is a distinction to be drawn between directly stimulating affective states, influencing affective states and monitoring affective states. Affective BCIs may be used for all three. This section addresses ethical issues that arise from the ability of affective BCIs to monitor affective states. The information gathered from this monitoring could subsequently be used to manipulate or induce affective states. However, even without the additional manipulation, the monitoring itself is in need of ethical scrutiny.

Similar to tracking sleep, exercise and one’s heartbeat via devices and apps, tracking affective states are no longer off limits. Not surprisingly, tracking people’s emotions will be of interest to parties with economic motivations (e.g., marketing research) and in areas where customer satisfaction is an important factor. There are already companies that use technology, like smart identification badges that monitor speech (Heath 2016), to observe the emotions of employees in order to increase performance or obedience at the workplace. Affective BCIs would open up new opportunities for this kind of employee tracking by making possible a more precise monitoring. Similar to other brain reading technologies, the monitoring of affective states raises questions concerning mental privacy because it potentially allows for the detection of mental states that the subject may not wish to share. The use of affective BCIs can be linked to the general ethical discussion regarding mental privacy and the monitoring of mental states. Here, the ethical evaluation of the implications of affective BCIs can fall back on existing contributions. For example, Mecacci and Haselager (2017) helpfully provide a framework for the assessment of the implications of brain reading for mental privacy. This framework may also be used to assess the ethical challenges concerning mental privacy when affective BCIs are used to monitor affective states.

Monitoring emotions is not limited to the workplace or other professional contexts. There are applications available for emotional self-tracking and so-called emotional self-quantification (e.g., *Mercuryapp,* or *EmotionSense*). Both self-tracking practices and self-quantification have some ethical and cultural implications that need to be addressed. Lupton (2015) suggests that apps that track people’s sexual behavior may foster normative stereotypes about sex. By analogy, it is not very far-fetched to suspect that affective BCIs may have similar implications in that they could foster stereotypes concerning emotions. Closely connected to stereotypes is the issue of biases. Some authors have already pointed out the problem of biases embedded in neural devices (Yuste et al. 2017). Bias is an ethical issue that pertains to all forms of BCIs. However, the particularly crucial aspect in the case of affective BCI is that there are potential biases regarding affective states. For example, people have biases about emotions that are based on gender or age (Fabes and Martin 1991). So, it is a sensible idea to make sure that biases concerning emotions are not embedded in affective BCI technology. Further, other authors have raised concerns regarding the disciplining effects of self-tracking and that self-tracking could infringe on values like autonomy and authenticity (Sharon 2017). The same concerns, then, need to be taken seriously regarding the tracking of affective states in general, and the tracking via affective BCI in particular.

This does not rule out that monitoring affective states via affective BCIs could enhance autonomy and contribute positively to one’s well-being. For example, neurofeedback has been shown to be a valuable aid in the regulation of brain areas responsible for emotions (Johnston et al. 2010). Especially affective BCIs that provide some feedback regarding the emotional states of the user may help to gain some control over these states. However, this puts another ethical issue into the spotlight: The possibility of affective BCI-systems with real-world applicability may put social pressure on some individuals to self-regulate their emotions with the help of affective BCIs in order to fall within the domain of what is considered affectively ‘normal’.

Using an affective BCI may also have some repercussions on the ability to reflect on and engage with emotions and for some people the potential comprehensive monitoring ability of affective BCIs may result in an alienation from their emotions. Recall the camera, described in the introduction, that automatically takes pictures whenever one is emotionally engaged or the sound system that plays music according to the mood one is in. In these two cases, there is only limited need for people to pay attention to their emotions and reflect on whether it is worthwhile to take a picture or to think about which music best suits their mood. The technology takes care of these decisions by automatically making the choice for the user. In cases like these, the ability to reflect on an emotion and deliberate whether to act on that emotion is compromised by the affective BCI. This reflection and deliberation, however, is a crucial component of being a moral agent. The role of affective states in human life, the ability of humans to notice, to control and to cultivate emotions in order to be a moral person has been a key issue of ethics throughout history. If people do not have to take care of their affective states because of affective BCIs, reconsiderations of relevant presumptions about human conduct could become necessary.

#### Affective BCIs and Influencing Affective States

Besides monitoring affective states, another relevant ethical issue that needs to be addressed is that affective BCIs can be utilized to influence affective states. This section addresses ways of influencing emotions that are not invasive. That means that the affective BCI system does *not* directly and invasively interfere with brain processes. Ethical issues that arise in connection with directly and invasively stimulating affective states in people are addressed in the next section. Please also note that the above-mentioned ethical challenges regarding monitoring of affective states may also play a role here because both influencing and directly stimulating affective states may rely on monitoring affective states in some form or other.

One possible way to influence affective states that affective BCIs could facilitate is nudging. Broadly speaking, nudging refers to interventions that influence people’s behavior without forcing them to commit a certain act (Sunstein 2015, 417). A familiar example is the fly in urinals that nudge users to aim at a certain spot. Another example is reminders or push notifications on smartphone applications. Digital technology is especially suited for a variety of forms of nudging that can respond flexibly to changes in user behavior. Affective BCIs seem to be optimal instruments for nudging, because decisions and emotions go hand in hand. Emotions contribute to the evaluations that people make and individuals usually take current and expected future emotions into account when they ponder a decision (Bagozzi et al. 2016; Mellers and McGraw 2001; Wilson and Gilbert 2005). Further, it is well established that emotions influence judgment and decision-making (Angie et al. 2011). In short, emotions shape intentions, decisions and actions. So, in many situations, influencing emotions of people means influencing their decisions and intentions and the actions that follow these intentions.

Technologies like affective BCIs allow for the manipulation of affective processes of humans. This intervention could infringe on the mental integrity of people. Mental integrity is the capacity of persons to have control over their mental states and brain data. This control entails that without consent nobody can monitor or manipulate these mental states or brain data (Lavazza 2018). Based on the ever-increasing technical ability to intervene in mental processes and the possible threat to mental integrity and cognitive liberty, some authors have argued for a legal protection of the mental realm (Bublitz and Merkel 2014). Future research should consider in more detail the potential implications of affective BCIs for mental integrity and cognitive liberty. Please note here that matters of cognitive liberty and mental integrity also apply for more direct forms of intervention in affective states, that are addressed in the next section.

Imagine an affective BCI-system that constantly reads the emotional state of the user. This kind of information is a valuable resource for companies and governments that are inclined to influence or nudge people to make certain economic or political decisions. Already today there seems to be increasing (mis)use of emotions in politics. Particularly the 2016 presidential election in the United States has brought into sharp focus the connection between technology and the manipulation of the feelings of voters. Artificial intelligence in the form of machine learning and social media was used to micro-target people in order to influence their emotions (Ghosh and Scott 2018; Polonski 2017). Some scholars even see the increasingly technologically mediated influence of emotions as a threat to democracy. For example, the historian Yuval Noah Harari cautions that because of the ability to manipulate emotions by advanced technology, ‘democratic politics will mutate into an emotional puppet show’ (Harari 2018, 68).

When affective BCIs are used in nudging schemes, well-known ethical issues of nudging come to the fore. Some authors have expressed the worry that nudging is detrimental to fairness and freedom (Goodwin 2012). Others have argued against these criticisms, for example by pointing out that nudging may promote autonomy if it steers behavior towards a direction that is in line with one’s own values and character (Sunstein 2015). Using affective BCIs in order to nudge people can be beneficial. Consider an affective BCI that has registered that the users are more inclined to use medication when they are in a certain affective mental state and, perhaps in collaboration with an ambient assisted living system, utilizes this information to nudge them to take their medicine. The benefits in this scenario are obvious. However, the same affective BCI may play a role in a scenario where information about the affective state of the users is used to influence them politically or to nudge them into buying certain goods. While noting that nudging is a complex ethical issue, it is nevertheless important to draw attention to whether and when it is ethically appropriate to use affective BCIs as nudging tools and whether affective BCI research should pursue designs that lend themselves to nudging.

Emotions play a crucial role in decision-making, and particularly in the evaluation of products and the decision to buy them. Coleman and Williams (2013) demonstrate how people’s social identity is connected to a specific emotion profile and that consumers prefer emotional messages that are compatible with their social identity. For example, when individuals are primed with their athlete identity, they find anger-based advertisement more persuasive because anger is consistent with the emotion profile of their social identity as athletes. Given the tight connection between consumer decisions and emotions, it is no surprise that companies want to get their hands on information about people’s emotions in order to target them. For example, Facebook has a history of influencing the emotions of its users. In a widely reported study, Facebook manipulated the news feed of users in order to assess the effect of this manipulation on their emotions (Kramer et al. 2014). Further, a recently leaked Facebook document includes the claim that the company’s algorithms can detect the emotional states of their users, allowing advertisers to determine the right moment when teenagers are in need of a ‘confidence boost’ (Levin 2017), which is another way of saying that they are a good target for advertising. Thinking even further, affective BCI allows for distinct access to the affective states of prospective customers, which in turn can be utilized to create input according to the emotion profile of particular individuals or to emotionally influence people in such a way that makes them more likely to buy a specific product.

Affective BCIs could be used to influence human emotions through an adjustment of that person’s environment. Consider this: As devices become more and more connected, and ambient living and the so-called internet of things become feasible, affective BCIs could in principle be connected to all kinds of devices and smart surroundings. For example, an affective BCI may alter the environment via an ambient lighting system (Andujar et al. 2015), either to match the affective state of the users or to influence their emotions. For instance, when an affective BCI user is angry, their apartment’s lighting could adjust automatically in order to help them calm down. In a scenario like this, the question may be raised about how much the person was actually in charge of the emotional regulation and how much of it was due to the smart interconnected environment. Ultimately, affective BCIs may prompt us to do the ‘symbolic labour’ (Schermer 2009, 221) of re-interpreting and re-conceptualizing the idea of responsibility for emotions.

Although responsibility ascription is usually limited to actions, there is a case to be made that people are also responsible for their emotions because they can be subjected to emotional self-regulation (Roberts 2015). Affective BCIs complicate this responsibility issue, because emotional self-regulation may (in part) be outsourced to the affective BCI-system, which raises the question of how much ‘self’ is actually involved in emotional regulation. As hinted at previously, techniques for controlling and regulating emotions are of fundamental ethical relevance and have played a crucial role in philosophy, psychology and psychotherapy (Charland 2007). New ways of technologically regulating emotions are ethically relevant and need much more consideration. It is prudent to get a head start and to think about these ethical (and conceptual) implications of plausible affective BCI applications before the technology is too far along and much of the ethical reflection is futile. Of course, for new and emerging technologies like affective BCIs it is hard to consider in advance the ethical and social implications. Even harder to grasp are the potential consequences of novel technologies for what it means to be human. Because of these difficulties we should be open to novel ways of exploring these issues. For example, Roeser et al. (2018) have demonstrated that art can be helpful in the ethical reflection on brain–computer interfaces. Extending this idea, one may expect that art will also serve us well in grasping the implications of affective BCIs.

#### Affective BCIs and Directly Stimulating Affective States

So far, the ethical aspects of indirectly (or non-invasively) influencing affective states with affective BCIs have been discussed. However, affective BCIs may also enable a more invasive and direct way to influence people’s affective states. Eliciting affective responses from people by means of brain stimulation requires ethical considerations.

It is already possible to directly stimulate affective states via invasive technology. For example, electric stimulation of the amygdala can induce negative emotions (e.g., fear) and happiness (Lanteaume et al. 2007). Although closed-loop brain stimulation is still in its early stages, it is conceivable to set up an affective BCI system as a closed-loop system. A closed-loop system receives continuous feedback from the brain and stimulates brain activity accordingly. So, a closed-loop affective BCI system would automatically stimulate specific brain areas in order to bring about or suppress certain affective states. This has ethically relevant implications: Closed-loop affective BCI systems put some pressure on the relation between emotional self-regulation and responsibility in that the machine, and not the user, does the regulating. Further, there is already a precedent when it comes to the possible negative effects of stimulating mental states with closed-loop systems. It has been argued that deep brain stimulation (DBS), that is a technique for sending electrical impulses to the brain via implants, may potentially undermine agency and personal identity (Goering et al. 2017) and that DBS could also lead to self-estrangement (Gilbert et al. 2017).

The technology of DBS could be problematic when it is used to directly stimulate affective states and people actually worry about what this technology does to their emotions. In interviews with participants of DBS trials, people expressed the concern that DBS could be used to bring forth emotions that are not authentic, thereby undermining their sense of self (Klein et al. 2016). In light of this, it seems worthwhile to accompany the development and implementation of affective BCI systems with an assessment of potentially sensitive issues. For instance, what happens when an affective BCI-induced emotion is in conflict with the evaluative judgment of the person? Even without affective BCIs these so-called recalcitrant emotions are a common occurrence. For instance, despite their belief that tiny dogs and flying are not dangerous at all, some people experience fear when they encounter small dogs or when they have to fly (phobias are a pervasive form of recalcitrant emotion). Further, people sometimes have recalcitrant bouts of anger or jealousy that conflict with their judgment about a situation. However, despite being a common occurrence, recalcitrant emotions can be a somewhat confusing experience. Further, not being able to differentiate whether an affective state originated from oneself or was triggered by the affective BCI system may be very disturbing. Provided that harm should be prevented whenever possible, the practical recommendation here seems to be to make sure that the potential for psychological distress is kept at a minimum. The real and potential power of affective BCIs to manipulate emotions calls for ethical scrutiny.

Although the impact of neurotechnology and BCI on the self and personhood has already received some attention (Fenton and Alpert 2008; Glannon 2016; Hildt 2015; Tamburrini 2009), the role of emotions in these issues needs to be considered more thoroughly. Emotions are important for a sense of self and personal identity. For instance, emotions play a crucial part in the constitution of autobiographical memories (Holland and Kensinger 2010). In turn, autobiographical memories are crucial for the constitution of the self and the sense of self (Prebble et al. 2013; Schechtman 1996, 2005). It seems then that the manipulation of emotions has a direct bearing on the constitution of the self. Given that affective BCIs can potentially aid such a manipulation, and given that emotions are a crucial aspect of what it means to be human, the possible consequences of this manipulation regarding the self, identity and personhood should not be taken lightly.

The military is one area where manipulating and stimulating affective states will likely play a crucial role. It is no secret that the military is very interested in using neurotechnology, including BCIs, for military purposes like vehicle control, military training and the enhancement of soldiers (Tennison and Moreno 2012). Specifically, influencing the affective states of soldiers has been said to have advantages, as it may help to ameliorate traumatic experiences after combat or attenuate emotions like anger, which could lead to atrocities (Beard et al. 2016). Further, soldiers are required to control their emotions and build so-called emotional fitness in order to become more resilient (Howell 2015). Affective BCIs could be another tool to achieve the goal of emotion control and emotional fitness in soldiers. Consider the possible uses of affective BCIs for the suppression of fear and empathy, or the use of affective BCIs to modulate anger. Military applications of neurotechnology and enhancement for military purposes involve a host of ethical issues (Beard et al. 2016; Moreno 2012) that also pertain to affective BCIs. For example, affective BCIs may be used to dampen certain emotions in soldiers (e.g., remorse, empathy, or fear) so that they are more aggressive and courageous. However, altering the emotions of soldiers in this way raises crucial questions of responsibility ascription and how much this interference affects moral decision-making.

## Conclusion

Although the development of affective BCIs is still at an early stage, concrete ethical issues can already be identified and should be discussed. Some ethical issues, like bodily harm or data security, are not new but pertain to all neurotechnologies. While acknowledging this, this paper went beyond these common issues and introduced with potential ethical issues that are particular to affective BCI technology. Specifically, the paper considered ethical concerns regarding monitoring, influencing and directly stimulating affective states.

Some use contexts of affective BCIs require a keener eye on the ethical issues than others. Generally, affective BCI technology appears to be less problematic when the applications do not involve a direct stimulation of affective states. Directly manipulating affective states is a bigger intervention into the mental set-up of a person, with potentially longer lasting consequences that may include changes that are irreversible. Further, scientific research and clinical applications seem to be the least problematic contexts for using affective BCIs because there are strict regulations and procedures that seek to limit harm as far as possible and that include informing people about the underlying technology and its risks and benefits. Nonetheless, some of the ethical concerns identified in this paper, like problems with false expectations or informed consent, are important in the clinical applications of affective BCI.

Although the majority of applications for affective BCIs are currently in clinical research and therapy, the future will likely see an increase in non-clinical applications. The use of affective BCI will be more problematic in contexts where people do not have a firm grasp on what is going on and what the technology does to them. This is usually the case in the context of consumer products with its lack of rigorous procedures regarding informed consent. To prevent misuse and abuse, the workings of the affective BCI should be as transparent as possible to the user. Unfortunately, if the past is any indication, making the workings of devices and systems transparent to people is not very high on the list of priorities of technology companies. To the contrary, new opportunities for the manipulation of people, either by companies or governments, are one of the greatest worries regarding affective BCI. For example, emotional profile building could help to subtly emotionally influence people for economic or political gain. Due to the sensible nature of data about mental states, issues of mental privacy, cognitive liberty and mental integrity have to be raised with stronger emphasis.

Humans have created multiple means to influence their minds. The list includes alcohol, synthetic drugs and various kinds of emotionally engaging entertainment. So it is no stretch of the imagination that people will one day willingly submit to the direct or indirect stimulation of their affective states for various recreational purposes. For example, affective BCIs could be used to stimulate affective states in order to enhance the experience of movies, musical performances or video games. The novel way of monitoring, influencing and stimulating affective states with BCI could have a deep impact on individuals and on society. These new techniques could influence emotional self-regulation, autobiographic memory, sense of self, identity, autonomy, authenticity and responsibility ascriptions. Further, for some individuals the availability of affective BCIs may create social pressure to use this technology to alter their affective states.

Because of the highly likely expansion of affective BCI technology into several non-clinical areas, it is important to scrutinize the various ethical implications of this technology as early as possible. This paper is a step in this direction.

## Footnotes

1 Neurocam relies on the headset developed by the company Neurosky (http://neurosky.com).
